# Dietary fats and serum lipids in relation to the risk of ovarian cancer: a meta-analysis of observational studies

**DOI:** 10.3389/fnut.2023.1153986

**Published:** 2023-09-14

**Authors:** Xu Zhang, Hong-Mei Ding, Li-Feng Deng, Guo-Chong Chen, Jie Li, Ze-Yin He, Li Fu, Jia-Fu Li, Fei Jiang, Zeng-Li Zhang, Bing-Yan Li

**Affiliations:** ^1^Department of Nutrition and Food Hygiene, School of Public Health, Medical College of Soochow University, Suzhou, China; ^2^Department of Obstetrics and Gynecology, The First Affiliated Hospital of Soochow University, Suzhou, China; ^3^Department of Obstetrics and Gynecology, The Second Affiliated Hospital of Soochow University, Suzhou, China; ^4^Department of Occupational and Environmental Health, School of Public Health, Medical College of Soochow University, Suzhou, China

**Keywords:** ovarian cancer, dietary fat, serum lipid levels, meta-analysis, observational study

## Abstract

Although numerous epidemiological studies investigated the association between dietary fat intakes or serum lipid levels and ovarian cancer risk, a consistent and explicit conclusion for specific dietary fats or serum lipids that increase the risk of ovarian cancer is not available. In this study, a systematic review and meta-analysis were conducted to assess the key dietary fats and serum lipids that increased the risk of ovarian cancer. Databases such as PubMed, Web of Science, and EMBASE were searched for observational studies. A total of 41 studies met the inclusion criteria, including 18 cohort and 23 case–control studies (109,507 patients with ovarian cancer and 2,558,182 control/non-ovarian cancer participants). Higher dietary intakes of total fat (RR = 1.19, 95% CI = 1.06–1.33, *I*^2^ = 60.3%), cholesterol (RR = 1.14, 95% CI = 1.03–1.26, *I*^2^ = 19.4%), saturated fat (RR = 1.13, 95% CI = 1.04–1.22, *I*^2^ = 13.4%), and animal fat (RR = 1.21, 95% CI = 1.01–1.43, *I*^2^ = 70.5%) were significantly associated with a higher risk of ovarian cancer. A higher level of serum triglycerides was accompanied by a higher risk of ovarian cancer (RR = 1.33, 95% CI = 1.02–1.72, *I*^2^ = 89.3%). This meta-analysis indicated that a higher daily intake of total fat, saturated fat, animal fat, and cholesterol and higher levels of serum triglycerides were significantly associated with an increased risk of ovarian cancer.

## 1. Introduction

Ovarian cancer is the most lethal gynecological malignancy, with limited screening modalities due to its low anatomical location, and lack of early symptoms and specific biomarkers. Additionally, 90% of reported ovarian cancer cases are epithelial ovarian cancer, which is already in advanced stages (stages III–IV) at the time of diagnosis and has a poor prognosis (1, 2).

Numbers of factors are suggested to impact the reproduction of ovarian cancer. High parity, oral contraceptive use, high lactation duration, tubal ligation, hysterectomy, oophorectomy, and salpingectomy are beneficial factors for ovarian cancer (3, 4). On the contrary, environmental endocrine disruptors, pelvic inflammation, and lipids are harmful factors in ovarian cancer (5–7), and a relationship between early adulthood and a high mortality rate in ovarian cancer patients may exist (8, 9). Particularly, omental metastasis, in which ovarian cancer cells lay over the omentum, an apron-like layer of adipose tissue in the peritoneal cavity, leads to significant consequences for patient morbidity and mortality (6).

Epidemiological evidence has gradually accrued that links obesity and dyslipidemia with higher rates of cancer and a higher risk of cancer-related mortality (9, 10). Specifically, diet fats might be a key modifiable factor, and serum lipids may be a potential predictor for diseases including cancer, both of which were associated with ovarian cancer risk and progression through altering systemic inflammation (11–13). A randomized controlled trial of 48,835 postmenopausal women revealed a 40% decrease in the incidence of ovarian cancer in individuals with a low-fat diet compared to a traditional diet (14). Moreover, some studies have reported that dietary polyunsaturated fat is a beneficial factor for ovarian cancer. For example, an Italian case–control study showed that n-3 polyunsaturated fatty acid was associated with a lower risk of ovarian cancer (15). Nevertheless, others suggested a lack of evidence for associations between dietary fat intake and ovarian cancer risk. A case–control study from Australia indicated no evidence for a protective role of n-3 polyunsaturated fatty acids in ovarian cancer (16). Meanwhile, higher concentrations of serum total cholesterol and high-density lipoprotein cholesterol (HDL-C) were reported to be associated with a lower overall risk of cancer (17). Higher serum cholesterol levels, however, were associated with a higher risk of ovarian cancer, according to nested case–control research from a serum bank (18).

Interpretation of epidemiological findings evaluating the relation between dietary fat and serum lipids and ovarian cancer may be hampered by differences in geographic dietary patterns and different characteristics of participants, as well as by adjustment for confounding factors such as total energy intake, BMI, or reproductive history. Therefore, quantitative analysis of the relationship between dietary fat, serum lipids, and ovarian cancer is essential and may also provide insight into the etiology of this complicating disease. Previously, epidemiological studies and meta-analyses have mostly focused on the association of single factors such as dietary fat or serum lipid levels with ovarian cancer risk (19–21). Currently, there are fewer meta-analyses that also summarize the association between dietary fat, serum lipid levels, and ovarian cancer risk. In this study, we performed an up-to-date and expanding meta-analysis to summarize epidemiological evidence from observational studies on both associations of dietary fat intakes and serum lipid levels with ovarian cancer risk by considering potential confounding factors using stratified sub-analyses and meta-regression analysis.

## 2. Materials and methods

The present study was conducted following the Meta-analysis of Observational Studies in Epidemiology (MOOSE) guidelines (22). This systematic review is registered in the PROSPERO International Prospective Register of Systematic Reviews with the identification code CRD42022349731.

### 2.1. Search strategy

An electronic database search was conducted to determine peer-reviewed articles published until 1 October 2022. Databases included PubMed, Web of Science, and Embase. The key search terms are dietary fat or fat intake, serum lipid, and ovarian cancer. The search strategy in the Supplementary Table S1. The bibliographies in the full retrieved articles and previously meta-analyses and related reviews were also searched for additional studies (9, 19, 21, 23).

### 2.2. Study selection

The Population, Intervention, Comparison, Outcome, and Study Design (PICOS) (24) criteria were used for the studies screened, as shown in Supplementary Table S2.

Studies that met the criteria below were included if they: (1) observational studies with cohort, case–control or nested case–control, (2) reported the outcome of interest as the occurrence of ovarian cancer or the incidence of tumors including ovarian cancer, (3) reported the exposure of interest as dietary fat intake (total fat, saturated fat, unsaturated fat, trans-fat, animal fat, plant fat, dairy fat, and cholesterol) or its subtypes or serum cholesterol level, and (4) reported odds ratios (ORs), relative risks (RRs), or hazard ratios (HRs) along with 95% CI to calculate.

### 2.3. Data extraction

Data extraction was completed independently by two authors, with any discrepancies resolved by discussion. Information extracted from each study included study characteristics (author, publication year and aria, study name, and sample size), amount or frequency of exposures (type and source of dietary fat, and serum lipids), RRs and 95% CI, and variable adjusted factors.

### 2.4. Quality and level of evidence assessments

The methodological quality of included studies was evaluated by the 9-star Newcastle-Ottawa Scale (NOS) scoring criteria (25). Literature selection, data extraction, and quality assessment were reworked and rechecked for accuracy by co-authors (Supplementary Tables S3, S4).

### 2.5. Analyses

The incidence of ovarian cancer was the outcome variable. We estimated the summary effect size, including RRs, ORs, or HRs and their corresponding 95% CIs for ovarian cancer among participants in the highest category of intake (dietary total fat and cholesterol, saturated fat, monounsaturated fat, polyunsaturated fat, trans-fat, animal fat, plant fat, and dairy fat) compared with the lowest category. Serum cholesterol, triglycerides, HDL-C, low-density lipoprotein cholesterol (LDL-C), and dyslipidemia, which reflect serum lipid levels, were also extracted for this study simultaneously. If there are multiple associated estimates, the most adjusted risk estimate was extracted. Of the included studies, ORs, RRs, and HRs were calculated using logistic regression analysis and Cox proportional hazard models. Because of the low absolute risk of ovarian cancer, all results of associations in these studies were reported as RRs, and the estimates of ORs or HRs from case–control studies and cohort studies were all assumed to be valid estimates of the RR.

Heterogeneity was evaluated by the *Q*-test and the *I*^2^ statistic. The significance level of the *Q-*test was defined as *p* < 0.10 and *I*^2^ values ≥ 50%, which indicated significant heterogeneity (26). Pooled effect sizes and their 95% CIs were estimated using a random effects model. Subgroup analyses from a pre-determined list of factors known to influence ovarian cancer risk (family history, oral contraceptive use, menopausal status, hormone use, pregnancy times, and body mass index), dietary assessment (total energy intake), and study characteristics (cohort vs. case–control studies, geographic location) were also conducted. The effects of lifestyle factors (smoking, alcohol consumption, and physical activity) were additionally synthesized, and fasting status were also incorporated. Heterogeneities between subgroups were evaluated by meta-regression analysis once again. The influence of individual studies was assessed by sensitivity analysis.

Funnel plots and Egger's test were used to assess publication bias (27), and “trim and fill” was also used to correct a bias (28). All statistical analyses were performed using STATA software, version 15.0 (STATA Corp, College Station, TX, USA).

## 3. Results

### 3.1. Study selection and characteristics

**Figure 1:**
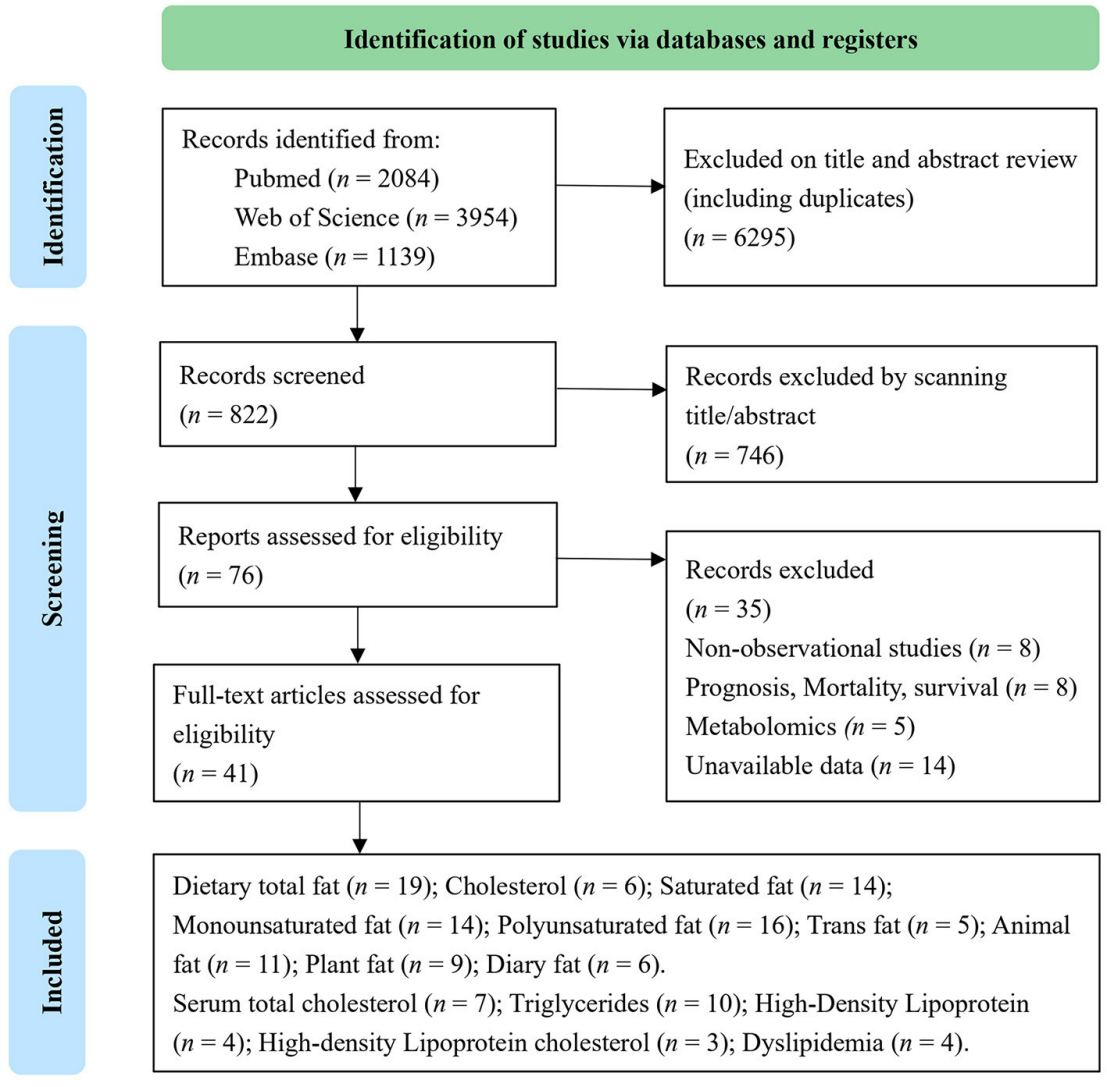
Flow diagram of the literature search process.

Out of 7,117 publications collected from the electronic and manual literature searches, 7,041 articles were excluded through browsing titles or abstracts. In the next step, 76 potentially relevant publications that appeared to meet the specified inclusion criteria were reviewed further. After the assessment of these full texts, 35 records were excluded due to insufficient data or the use of duplicate samples in these studies. Finally, 41 studies were considered eligible and underwent quantitative evaluation (Figure 1). Among selected research studies, nine cohort studies (29–37) and 17 case–control studies (15, 16, 38–52) focused on the types and sources of dietary fat, including dietary total fat, cholesterol, saturated, monounsaturated, polyunsaturated, trans-fats, animal, plant, and dairy fats (Table 1). Meanwhile, 18 cohort studies (18, 53–60) and six case–control studies (62–67) focused on serum lipids, including total cholesterol, triglycerides, HDL-C, LDL-C, and dyslipidemia (Table 2). A flow chart of study selection is displayed in Figure 1.

**Table 1:** Characteristics of 18 cohort studies included in the meta-analysis of dietary fat intake and serum lipid levels to the risk of ovarian cancer.

**References**	**Locale**	**Study name and design**	**Sample size: case/total**	Type of variables^*^	**Exposure categories (dietary assessment)**	**RR (95% CI) *P***	Adjusted confounding factors^**^
Rice et al. (29)	U.S.A.	Nurses' Health Study (NHS) cohort	700/121,700	Total fat (g/d)	Q4 vs. Q1	1.25 (0.95–1.64) 0.08	a, b, c, d, e, f, g, h, j, k, o
				Cholesterol (mg/d)		1.35 (1.08–1.69) 0.01,	
				Saturated fat (g/d)		1.35 (0.97–1.88) 0.05	
				Trans-fat (g/d)		0.82 (0.61–1.10) 0.13	
				Monounsaturated fat (g/d)		0.98 (0.68–1.40) 0.80	
				Polyunsaturated fat (g/d)		1.25 (0.96–1.63) 0.18	
				Animal fat (g/d)		1.57 (1.20–2.06) < 0.01	
				Plant fat (g/d)		1.17 (0.92–1.48) 0.36	
Merritt et al. (30)	European	European Prospective Investigation into Cancer and Nutrition	1,191/325,007	Total fat (g/d)	Q4 vs. Q1	1.16 (0.96–1.40) 0.05	a. b. e. f. h. k
				Cholesterol (mg/d)		1.24 (0.97–1.58) 0.12	
				Saturated fat (g/d)		1.17 (0.97–1.40) 0.15	
				Monounsaturated fat (g/d)		1.16 (0.93–1.44) 0.17	
				Polyunsaturated fat (g/d)		1.22 (1.02–1.48) 0.02	
				Animal fat (g/d)		0.96 (0.80–1.15) 0.86	
				Plant fat (g/d)		1.22 (0.98–1.52) 0.09	
Blank et al. (31)	Netherlands	NIH-AARP Diet and Health Study	695/151,552	Total fat (g/d)	Q5 vs. Q1	1.28 (1.01–1.63) 0.04	a, b, c, d, f, g, h, l, m
				Saturated fat (g/d)		1.03 (0.71–1.50) 0.98	
				Monounsaturated fat (g/d)		1.01 (0.63–1.60) 0.87	
				Polyunsaturated fat (g/d)		1.28 (0.92–1.77) 0.09	
				Animal fat (g/d)		1.30 (1.02–1.66) 0.03	
				Plant fat (g/d)		1.00 (0.79–1.27) 0.96	
Gilsing et al. (32)	Netherlands	Case–Cohort analysis	340/2,161	Total fat (g/d)	Q5 vs. Q1	1.01 (0.90–1.13) 0.80	a, b, f, h
				Trans-fat (g/d)		1.14 (1.03–1.28) 0.01	
				Saturated (g/d)		1.21 (1.04–1.41) 0.14	
				Monounsaturated (g/d)		0.85 (0.80–1.12) 0.44	
				Polyunsaturated (g/d)		0.90 (0.79–1.03) 0.33	
				Plant fat (g/d)		0.93 (0.81–1.07) 0.05	
				Animal fat (g/d)		1.11 (0.99–1.25) 0.19	
				Dairy fat (g/d)		1.13 (1.01–1.27) 0.17	
Chang et al. (33)	U.S.A.	California Teachers Study	280/97,275	Total fat (g/d)	Q5 vs. Q1	0.85 (0.58–1.24)	a, b, e, f, g, h, i, j, l
				Saturated fat (g/d)		0.72 (0.48–1.08)	
Kiani et al. (34)	U.S.A.	Adventist Health Study (United States)	71/13,281	Dairy fat (g/d)	T3 vs. T1	0.94 (0.70–1.27) 0.69	a, b, d, e, h
Mommers et al. (35)	Netherlands	Netherlands Cohort Study on Diet and Cancer	252/2,216	Dairy fat (≥31.0 g/d)	Q5 vs. Q1	1.53 (1.00–2.36) 0.11	a, f, h, j, n
Bertone et al. (36)	U.S.A.	Nurses' Health Study (NHS) cohort	358/80,258	Total fat (g/d)	Q5 vs. Q1	1.03 (0.72–1.45) 0.97	a, e, f, g, h, j, o
				Cholesterol/(mg/d)		1.08 (0.76–1.53) 0.63	
				Saturated fat (g/d)		0.91 (0.62–1.32) 0.97	
				Monounsaturated fat (g/d)		1.07 (0.75–1.52) 0.99	
				Polyunsaturated fat (g/d)		1.14 (0.79–1.63) 0.74	
				Trans-fat (g/d)		1.03 (0.72–1.47) 0.87	
				Animal fat (g/d)		0.95 (0.66–1.38) 0.97	
				Plant fat (g/d)		0.98 (0.68–1.43) 0.91	
				Dairy fat (g/d)		1.06 (0.73–1.54) 0.93	
Kushi et al. (37)	U.S.A.	Lowa Women's Health Study	139/29,083	Total fat (g/d)	Q4 vs. Q1	0.80 (0.47–1.36) 0.34	a, b, c, e, h, i, j, m, o
				Cholesterol (mg/d)		1.15 (0.90–2.67) 0.06	
				Saturated fat (g/d)		1.17 (0.69–1.97) 0.89	
				Monosaturated fat (g/d)		0.65 (0.38–1.13) 0.24	
				Polyunsaturated fat (g/d)		0.63 (0.38–1.03) 0.18	
				Animal fat (g/d)		0.98 (0.57–1.69) 0.56	
				Plant fat (g/d)		0.75 (0.44–1.27) 0.20	
Trabert et al. (53)	U.S.A.	(NHS) and NHSII cohorts and a longitudinal analysis in the UK Biobank.	290/32,826	Total cholesterol (mg/dL);	Q4 vs. Q1	0.81 (0.65–1.01) 0.06	a, d, e, f, g, h, k, o, p q
				Triglycerides (mg/dL)		1.12 (0.88–1.43) 0.87	
				HDL-C (mg/dL);		0.87 (0.70–1.07) 0.19	
				LDL-C(mg/dL);		0.89 (0.70–1.11) 0.12	
Shin et al. (54)	Korea	Retrospective Population-based Cohort Study.	56,682/288,119	Dyslipidemia	NA	1.40 (0.90–2.17)	a, d, j, s, r
Kabat et al. (55)	U.S.A.	Women's Health Initiative	115/16,366	Total cholesterol (10 mg/dL)	Q4 vs. Q1	1.62 (0.83–3.15) 0.29	a, d, e, f, g, h, i, l, j, m, t
				Triglycerides (10 mg/dL)		1.07 (0.55–2.05) 0.42	
				HDL-C(10 mg/dL)		0.56 (0.28–1.11) 0.10	
				LDL-C (10 mg/dL)		1.30 (0.66–2.57) 0.43	
Wu et al. (56)	Japan	Nested Case–Control Study	30/11,258	Total cholesterol (mg/dL)	NA	0.90 (0.26–3.13)	a, d, e, f, g, h, j, m, u
				Triglyceride (mg/dL)		1.16 (0.52–2.60)	
Strohmaier et al. (57)	Norway, Austria, and Sweden	Metabolic Syndrome and Cancer (Me-Can) project	733/289,273	Total cholesterol (mg/dL)	Q5 vs. Q1	1.27 (0.86–1.89) 0.12	a, b, d, g, h, j, k, q
Melvin et al. (58)	Swedish	Swedish AMORIS database	808/234,494	Total cholesterol (mmol/L)	Q4 vs. Q1	1.07 (0.85–1.38) 0.68	e, h
				Triglycerides (mmol/L)		0.93 (0.75–1.17) 0.31	
				LDL-C (mmol/L)		0.95 (0.53–1.71) 0.94	
				HDL-C (mmol/L)		0.95 (0.54–1.67) 0.81	
Bjorge et al. (59)	Norway, Austria, and Sweden	Metabolic Syndrome and Cancer (Me-Can) project	1,032/290,000	Total cholesterol (mmol/l)	Q5 vs. Q1	1.51 (0.98–2.32) 0.12	a, d, e, j, k
				Triglycerides (mmol/l)		1.12 (0.65–1.91) 0.81	
Borena et al. (60)	Norway, Austria, and Sweden	Metabolic Syndrome and Cancer (Me-Can) project	15,686/256,512	Triglycerides	Q5 vs. Q1	2.23 (1.93–2.78)	a, d, k, j
Helzlsouer et al. (61)	U.S.A.	Nested Case–Control Study	35/67	Total cholesterol	Cholesterol ≥ 230.67 mg/d	3.23 (0.9–11.3) 0.1	a, l, p, q, e

^*^HDL-C, high-density lipoprotein cholesterol; LDL-C, low-density lipoprotein cholesterol. ^**^Adjusted confounding factors, a, age; b, energy-adjusted intake; c, family history of ovarian cancer; d, body mass index; e, menopausal status at enrollment; f, oral contraceptive use; g, hormone therapy use; h, pregnancy times; i, physical activity; j, smoking status, pack-years of smoking, alcohol intake; k, calendar year; l, race; m, education n, dairy products, o, tubal ligation, p, blood draw time, q, fasting status, r, diabetes mellitus, and hypertension, s, income, t, age at menarche, u, waist-to-hip ratio.

**Table 2:** Characteristics of 23 case–control studies included in the meta-analysis of dietary fat intake and serum lipid levels to the risk of ovarian cancer.

**References**	**Locale**	**Study design**	**Sample size: case/control**	Type of variables^*^	**Exposure categories (dietary assessment)**	**RR (95% CI) *P***	Adjusted confounding factors^**^
Merritt et al. (30)	U.S.A.	New England Case–Control Study	1,872/1,978	Total fat (g/d)	Q4 vs. Q1	1.07 (0.89–1.29) 0.30	a, b e, f, h
				Cholesterol (mg/d)		0.97 (0.81–1.18) 0.93	
				Saturated fat (g/d)		1.11 (0.92–1.34) 0.26	
				Trans-fat (g/d)		1.30 (1.08–1.57) 0.02	
				Monounsaturated fat (g/d)		0.96 (0.80–1.15) 0.73	
				Polyunsaturated fat (g/d)		0.77 (0.64–0.94) 0.02	
				Animal fat (g/d)		1.04 (0.87–1.26) 0.27	
				Plant fat (g/d)		0.98 (0.81–1.17) 0.88	
				Dairy fat (g/d)		0.95 (0.79–1.14) 0.28	
Ibiebele et al. (16)	Australia	Case–Control Study	1,366/1,414	Polyunsaturated fat (g/d)	Q4 vs. Q1	0.78 (0.60–1.00) 0.04	a, b, m, d, j, f; h, e, g
Hu et al. (39)	Canada	Case–Control Study	442/5,039	Trans-fat (g/d)	Q4 vs. Q1	1.04 (0.68–1.58) 0.84	a, d, e, h, j, m
Chiaffarino et al. (40)	Italy	Case–Control Study	750/2,411	Monounsaturated fat (g/d)		0.80 (0.66–0.96)	c, f, h, m
				Polyunsaturated fat (g/d)		0.96 (0.76–1.21)	
Pan et al. (42)	Canada	Case–Control Study	442/2,135	Total fat (g/d)		1.21 (0.88–1.65)	a, b, d, e, h, i, j, m
				Saturated fat (g/d)		1.06 (0.78–1.45)	
				Monounsaturated fat (g/d)		1.26 (0.92–1.72)	
				Polyunsaturated fat (g/d)		1.28 (0.94–1.76)	
Tavani et al. (15)	Switzerland	Case–Control Study	1,031/2,411	Polyunsaturated fat (g/d)	Q4 vs. Q1	0.85 (0.77–0.93)	a, b, d, b, h, m
McCann et al. (43)	U.S.A.	Case–Control Study	124/696	Total Fat (g/d)	Q5 vs. Q1	1.51 (0.57–4.02)	a, b, e, h, f, m
				Cholesterol (mg/d)		1.46 (0.68–3.15)	
				Saturated fat (g/d)		1.46 (0.68–3.15)	
				Monounsaturated fat (g/d)		1.77 (0.73–4.31)	
				Polyunsaturated fat (g/d)		0.63 (0.28–1.41)	
Zhang et al. (41)	China	Case–Control Study	254/652	Animal fat (g/d)	Q4 vs. Q1	4.55 (2.2–9.3)	a, b, c, d, e, f, h, j, m, o, s
Salazar-Martinez et al. (44)	Mexico	Case–Control Study	84/629	Total fat	T3 vs. T1	0.60 (0.33–1.06) 0.06	a, b, h, i, r
				Cholesterol		0.53 (0.30–0.98) 0.04	
				Saturated fat		0.56 (0.31–1.02) 0.06	
				Monounsaturated fat		0.54 (0.30–0.99) 0.04	
				Polyunsaturated fat		0.61 (0.34–1.11) 0.07	
				Animal fat		0.66 (0.37–1.19) 0.16	
				Plant fat		0.81 (0.46–1.45) 0.48	
Risch et al. (46)	U.S.A.	Case–Control Study	641/564	Total fat	≥ 29.87 vs. < 19.17 g/d	1.16 (0.86–1.57) 0.32	a, b, h, f
				Cholesterol		1.15 (1.02–1.30) 0.02	
				Saturated fat		1.20 (1.03–1.40) 0.08	
				Monounsaturated fat		1.07 (0.90–1.27) 0.43	
				Polyunsaturated fat		0.86 (0.69–1.07) 0.19	
Tzonou et al. (47)	Greece	Case–Control Study	189/200	Total fat (g/d)	Q6 vs. Q1	0.97 (0.76–1.24) 0.83	a, e, h, j
				Cholesterol (mg/d)		1.19 (0.96–1.48) 0.10	
				Saturated fat (g/d)		1.17 (0.88–1.55) 0.28	
				Monounsaturated fat (g/d)		0.86 (0.71–1.05) 0.15	
				Polyunsaturated fat (g/d)		0.95 (0.77–1.17) 0.62	
Shu et al. (48)	China	Case–Control Study	172/172	Animal fat (g/d)	Q4 vs. Q1	1.70 (1.20–4.20) 0.07	m
				Plant fat (g/d);		0.80 (0.40–1.40) 0.58	
Slattery et al. (49)	U.S.A.	Case–Control Study	85/492	Total fat	T3 vs. T1	1.30 (0.70–2.30)	a, d, h
				Saturated fat		1.30 (0.60–2.60)	
				Monounsaturated fat		1.30 (0.70–2.30)	
				Polyunsaturated fat		1.20 (0.60–2.30)	
Lavecchia et al. (50)	Italy	Case–Control Study	455/1,385	Total fat	High vs. low	2.14 (1.59–2.88)	a
Cramer et al. (51)	U.S.A.	Case–Control Study	215/215	Animal fat	≥ 225 vs. < 125 intake	1.83 (1.00–3.38)	a, h, l,
Webb et al. (45)	Australia	Case–Control Study	824/1,132	Total fat	NA	1.86 (1.03–3.37)	a, b, d, f, h, j, m
Byers et al. (52)	U.S.A.	Case–Control Study	274/1,034	Total fat	NA	1.25 (0.90–1.73)	N/A
Staples et al. (62)	U.S.A.	Case–Control Study	593/752	Dyslipidemia	NA	0.61 (0.47, 0.80)	a, c, d, f, h, m, r
Michels et al. (63)	U.S.A.	Case–Control Study	16,850/281,878	Triglycerides	NA	1.05 (1.01–1.08)	a, f, j, l
Zeleznik et al. (67)	U.S.A.	Case–Control Study	252/252	Triglycerides	90th vs. 10th percentile	1.96 (1.20–3.20)	a, e, k, p, q
Chen et al. (64)	China	Case–Control Study	573/1,146	Triglycerides	≥1.70 mmol/L	2.86 (1.04–7.87)	e, g, h, s
				HDL-C	≥1.0 mmol/L	0.47 (0.38–0.58)	
Parazzini, et al. (65)	Italy	Case–Control Study	971/2,758	Hyperlipidemia	T3 vs. T1	0.86 (0.82–0.89)	a, e, h, m r
Bodmer, et al. (66)	U.K.	Case–Control Study	1611/9170	Dyslipidemia	NA	1.14 (0.95–1.35)	NA

^*^HDL-C, high-density lipoprotein cholesterol; LDL-C, low-density lipoprotein cholesterol. ^**^Adjusted confounding factors: a, age; b, energy-adjusted intake; c, family history of ovarian cancer; d, body mass index; e, menopausal status at enrollment; f, oral contraceptive use; g, hormone therapy use; h, pregnancy times; i, physical activity; j, smoking status, pack-years of smoking, alcohol intake; k, calendar year; l, race; m, education n, dairy products, o, tubal ligation, p, blood draw time, q, fasting status, r, diabetes mellitus, and hypertension, s, income, t, age at menarche, u, waist-to-hip ratio.

Eligible studies were published from 1989 to 2021 in North America (*n* = 20), Europe (*n* = 14), East Asia (*n* = 5), and Australia (*n* = 2). In these studies, a total of 2,667,689 individual data (109,507 patients with ovarian cancer and 2,558,182 control/non-ovarian cancer participants) were reviewed.

### 3.2. Dietary fat intake and ovarian cancer risk

**Figure 2:**
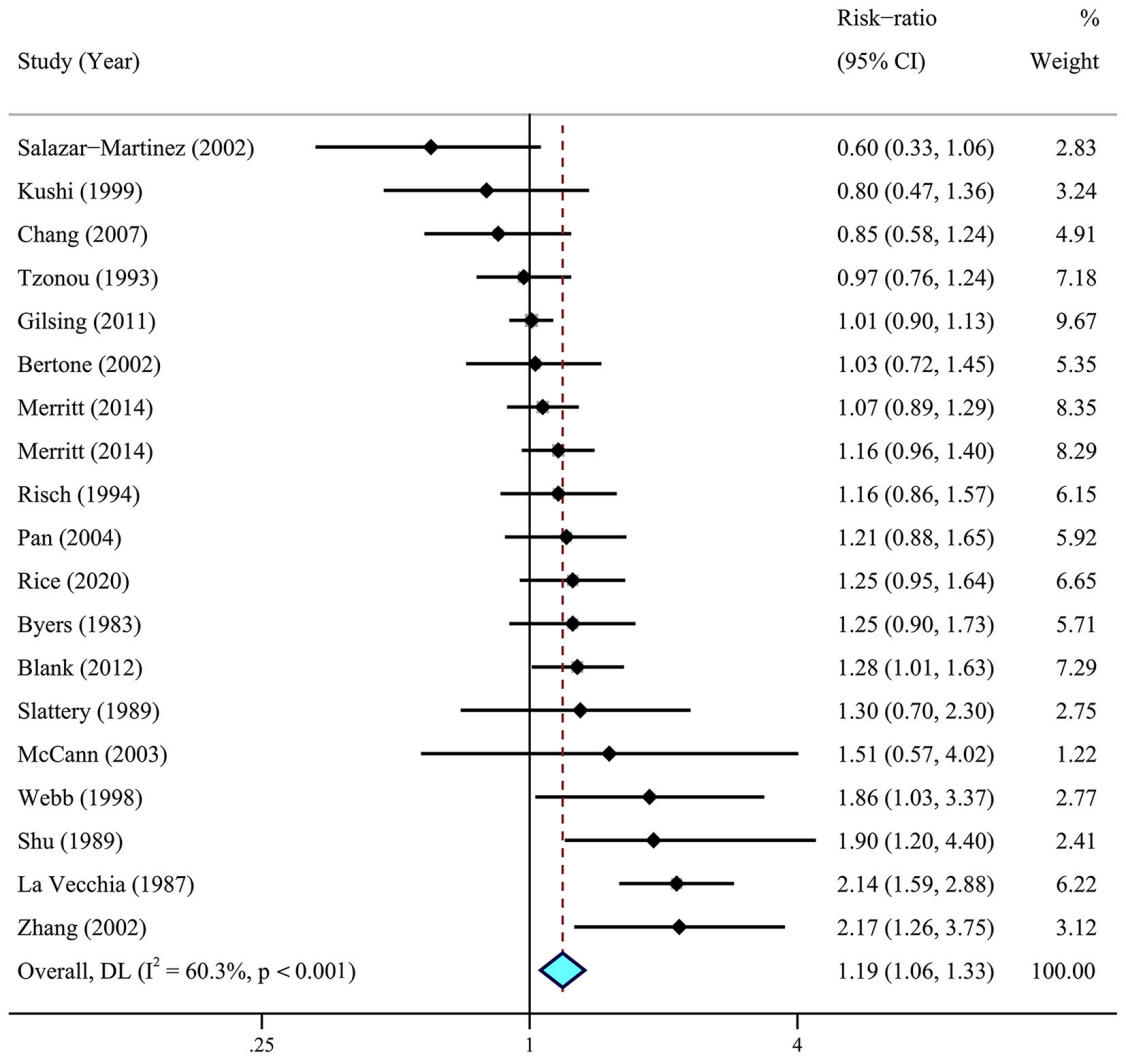
Relationship between dietary total fat intake and ovarian cancer risk. Effect estimate for random-effect analysis; *p*-value is for Cochran's *Q* statistics for heterogeneity; *I*^2^ is the proportion of total variation in study estimates from heterogeneity rather than sampling error.

**Figure 3:**
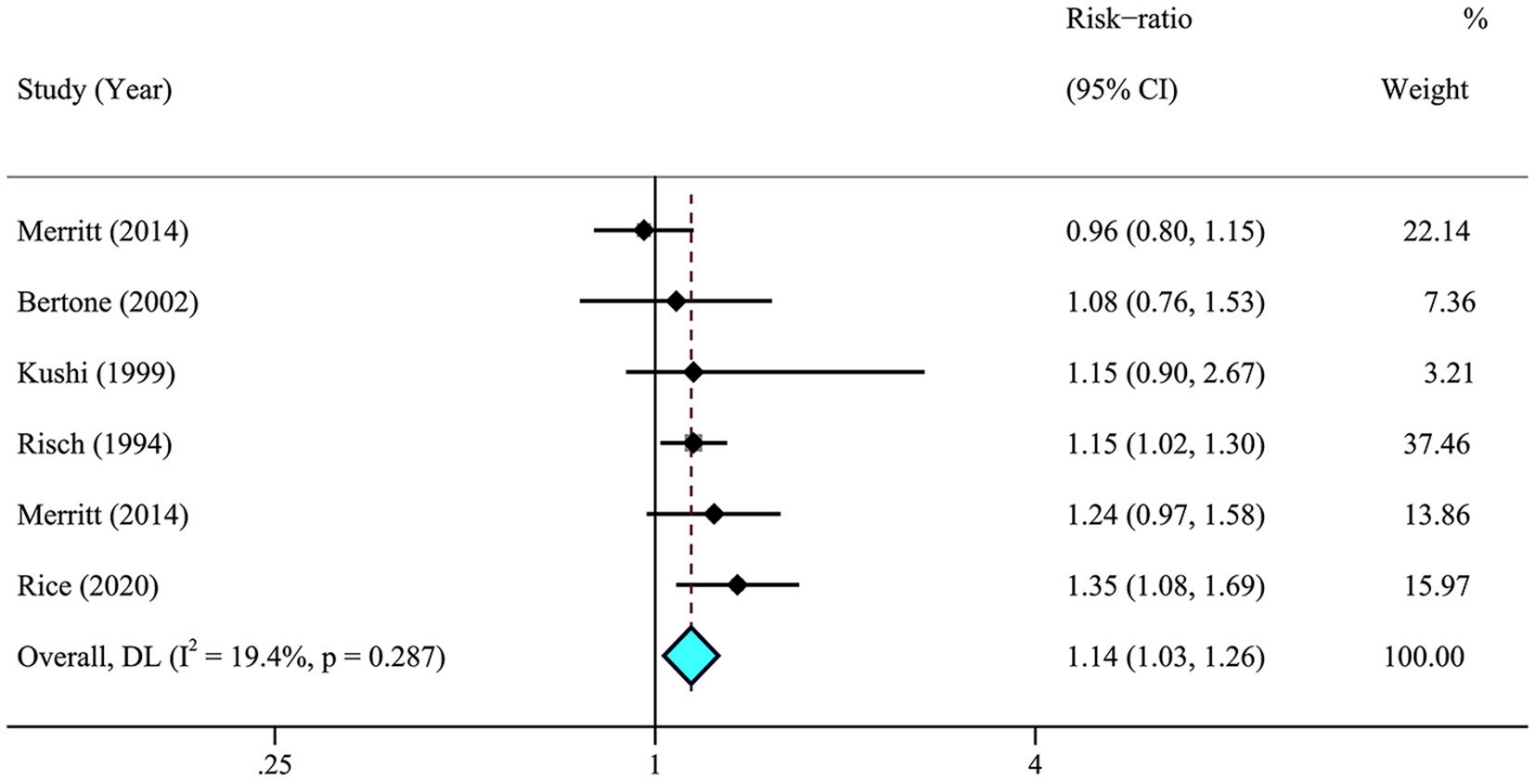
Relationship between dietary cholesterol intake and ovarian cancer risk. Effect estimate for random-effect analysis; *p*-value is for Cochran's *Q* statistics for heterogeneity; *I*^2^ is the proportion of total variation in study estimates from heterogeneity rather than sampling error.

A meta-analysis of 26 articles (29–33, 36, 37, 41–44, 46, 63) including 856,557 individuals was performed to assess the association between dietary fat intake and the risk of ovarian cancer. The results showed that individuals with higher total dietary fat (RR = 1.19, 95% CI = 1.06–1.33) (Figure 2) and cholesterol (RR = 1.14, 95% CI = 1.03–1.26, *I*^2^ = 19.4%) intake experienced a significantly higher risk of developing ovarian cancer (Figure 3). There was moderate heterogeneity across these studies for total fat (*I*^2^ = 60.3%, *p* = 0.009).

**Figure 4:**
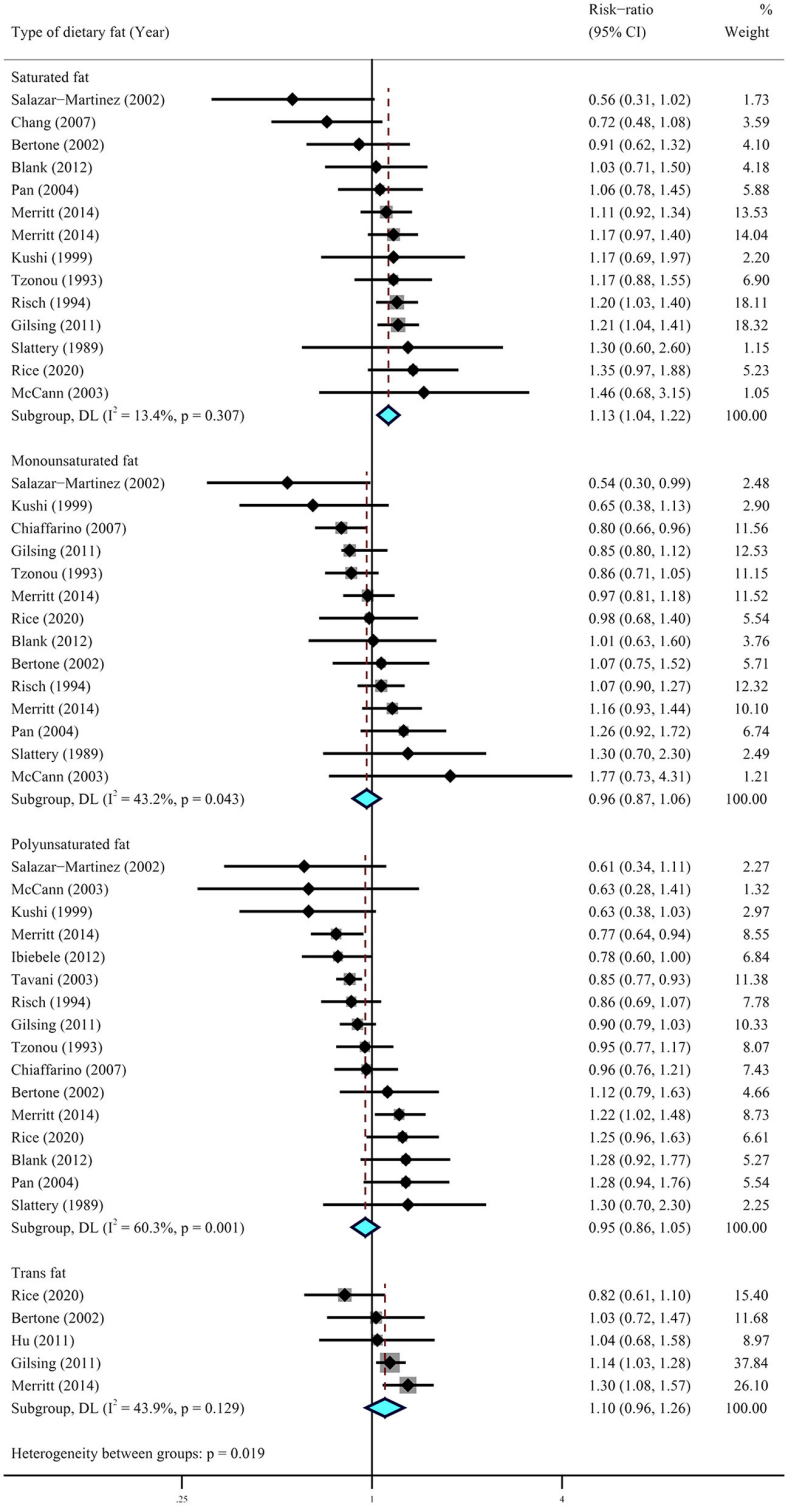
Summary most adjusted relative risks of saturated fat, monounsaturated fat, polyunsaturated fat, and trans-fat and ovarian cancer. Effect estimate for random-effect analysis; *p*-value is for Cochran's *Q* statistics for heterogeneity; *I*^2^ is the proportion of total variation in study estimates from heterogeneity rather than sampling error.

A further meta-analysis for the impact of specific types of dietary fat on ovarian cancer was conducted using data from six cohort studies (29–33, 36) and 12 case–control studies (15, 16, 39, 40, 42–44, 46, 47, 49). The results showed that higher dietary intake of saturated fat (RR = 1.13, 95% CI = 1.04–1.22, *I*^2^ = 13.4%) was statistically significantly associated with a higher risk of ovarian cancer. No significant association was observed for the dietary intake of monounsaturated fat (RR = 0.96, 95% CI = 0.87–1.06, *I*^2^ = 43.2%), polyunsaturated fat (RR = 0.95, 95% CI = 0.86–1.05, *I*^2^ = 60.3%), and trans-fat (RR = 1.10, 95% CI = 0.96–1.26, *I*^2^ = 43.2%) (Figure 3). Of note, the results from case–control studies showed that individuals who experienced a lower risk of ovarian cancer were significantly associated with higher intake of polyunsaturated fat (RR = 0.88, 95% CI = 0.80–0.97, *I*^2^ = 33.0%) and trans-fat (RR = 1.25, 95% CI = 1.06–1.49, *I*^2^ = 0.00%) with little heterogeneity (Figure 4).

**Figure 5:**
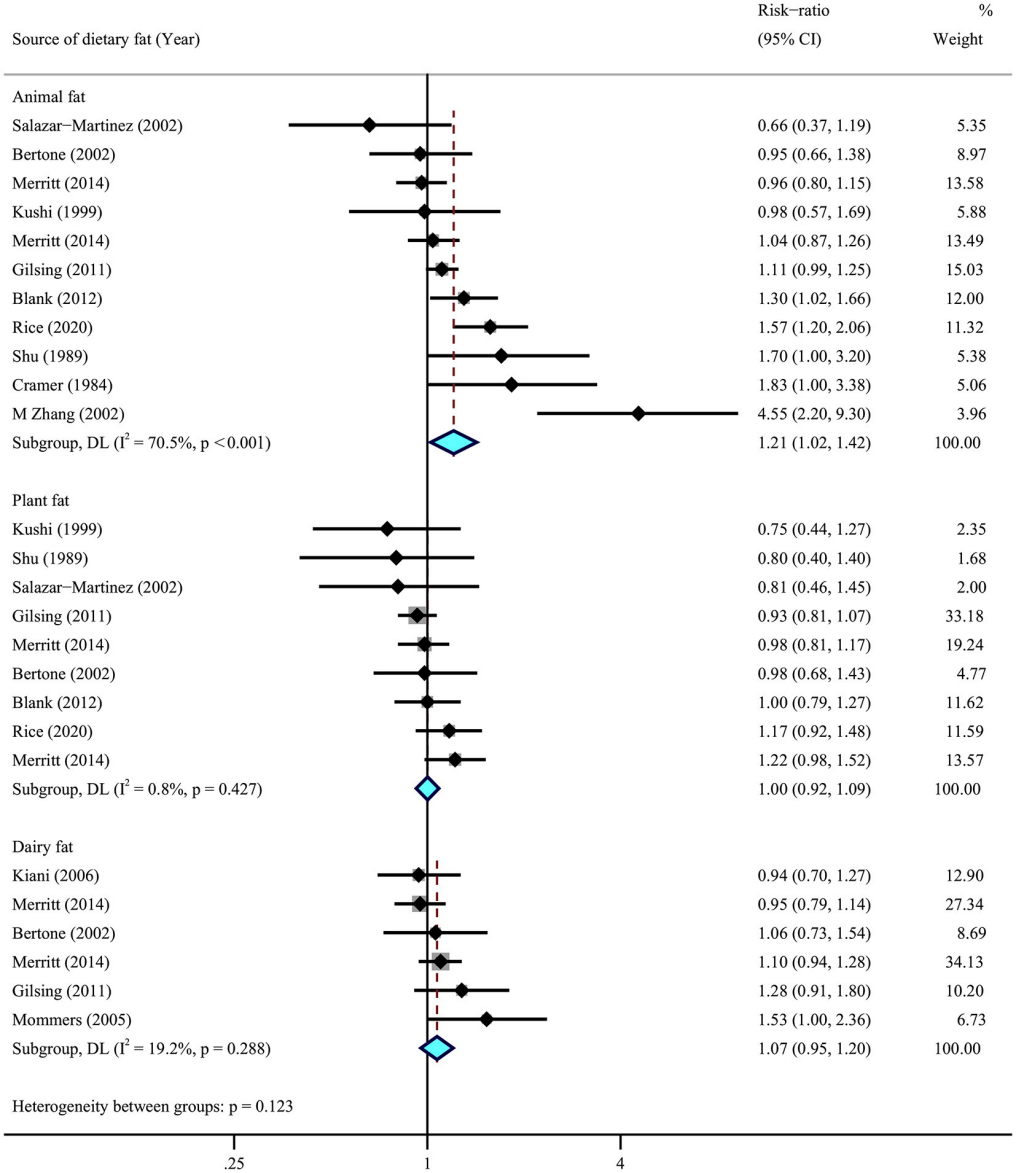
Summary most adjusted relative risks of animal fat, plant fat, and dairy fat and ovarian cancer. Effect estimate for random-effect analysis; *p*-value is for Cochran's *Q* statistics for heterogeneity; *I*^2^ is the proportion of total variation in study estimates from heterogeneity rather than sampling error.

We then conducted a meta-analysis for the sources of dietary fats, using data from eight cohorts (29–32, 34–37) and five case–control (38, 41, 44, 48, 51) studies. The results suggested that higher dietary intake of animal fat (RR = 1.21, 95% CI = 1.01–1.43, *I*^2^ = 70.5%) was significantly associated with a higher risk of ovarian cancer, while no association was observed for plant fat (RR = 1.00, 95% CI = 0.92–1.09, *I*^2^ = 0.8%) and dairy fats (RR = 1.07, 95% CI = 0.95–1.20, *I*^2^ = 19.2%) (Figure 5).

### 3.3. Serum lipid levels

**Figure 6:**
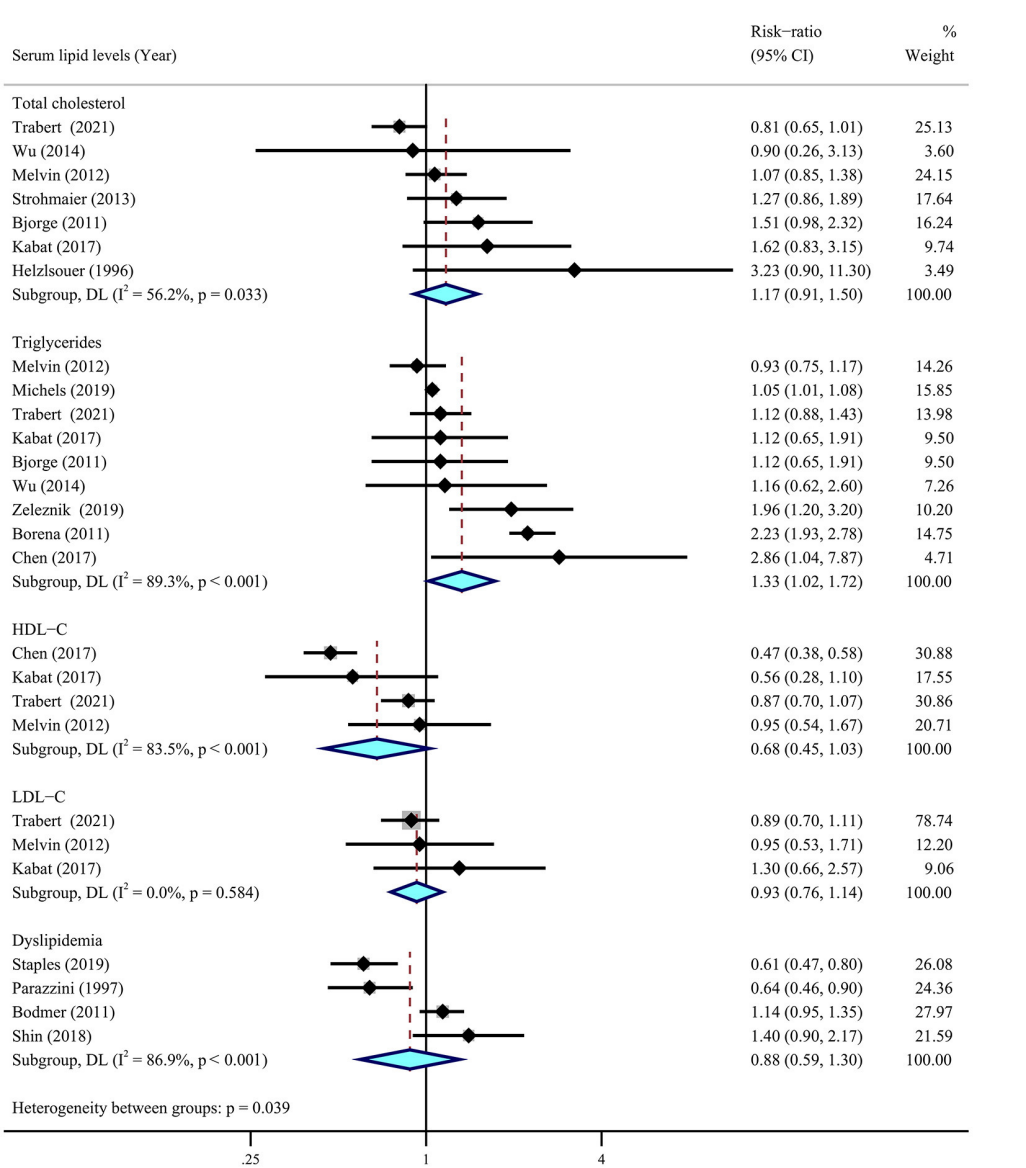
Relationship between serum lipid levels and ovarian cancer risk. Summary most adjusted risk-ratio of total cholesterol, triglycerides, high-density lipoprotein cholesterol (HDL-C), low-density lipoprotein cholesterol (LDL-C), dyslipidemia, and ovarian cancer risk. All effect estimate is random-effect analysis; *p*-value is for Cochran's *Q* statistics for heterogeneity; *I*^2^ is the proportion of total variation in study estimates from heterogeneity rather than sampling error.

Among selected nine cohort (18, 53–60) and six case–control (62, 63, 65–68) studies including 1,811,132 individuals, only seven studies showed a statistical association with the risk of ovarian cancer (Figure 6). The meta-analysis suggested a significant association between higher levels of serum triglycerides and a higher risk of ovarian cancer (RR = 1.33, 95% CI = 1.02–1.72, *I*^2^ = 89.3%), whereas there were insignificant correlations between serum cholesterol (RR = 1.17, 95% CI = 0.91–1.50, *I*^2^ = 56.2%), LDL-C (RR = 0.93, 95% CI = 0.76–1.14, *I*^2^ = 0.0%), and ovarian cancer risk. There were no obvious correlations between dyslipidemia and the risk of ovarian cancer (RR = 0.88, 95% CI = 0.59–1.30, *I*^2^ = 86.9%). In addition, there was a suggestive inverse association for HDL-C (RR = 0.68, 95% CI = 0.45–1.03, *I*^2^ = 83.5%) with ovarian cancer risk.

### 3.4. Sensitivity and subgroup analysis

We further excluded individual studies for these five significant associations observed between dietary total fat, cholesterol, saturated fat, animal fat, and serum triglycerides, which showed no change in combined relative risk (Supplementary Tables S1–S5). Moreover, sensitivity analyses did not suggest any significant associations with other dietary fats and serum lipids.

Stratified sub-analyses showed that among five significant associations to ovarian cancer risk, the higher heterogeneity in dietary total fat (RR = 1.19, 95% CI = 1.06–1.33, *I*^2^ = 60.3%) may be explained by differences in study type (case–control study, RR = 1.31, 95% CI = 1.08–1.58, *I*^2^ = 66.7%), area (Europe, RR = 1.23, 95% CI = 0.98–1.54, *I*^2^ = 83.4%), and total energy intake (no adjusted, RR = 1.45, 95% CI = 1.14–1.85, *I*^2^ = 67.1%) (Supplementary Table S5). Otherwise, the results of subgroup analysis were hardly modified by area, or various confounding factors, such as adjustments for BMI, menopausal status, oral contraceptives, hormone therapy, pregnancy times, and lifestyle factors (Supplementary Table S6).

### 3.5. Publication bias

There was no publication bias by means of visual inspection of funnel plots and formal statistical tests. None of the studies had a significant effect on the pooled risk estimates and 95% CIs (Supplementary Figures S6–S10). We further used Egger's test to check for potential publication bias for specific fats. No evidence of publication bias for dietary total fat (*p* = 0.963), cholesterol (*p* = 0.451), monounsaturated fat (*p* = 0.396), polyunsaturated fat (*p* = 0.135), trans-fat (*p* = 0.168), animal fat (*p* = 0.898), plant fat (*p* = 0.590), and dairy fat (*p* = 0.627) was shown. However, there was publication bias for saturated fat according to Egger's test (*p* = 0.009), and we re-estimated the effect size using the meta-trim method. The adjusted pooled effects did not alter the combined relative risk (RR = 1.13, 95% CI = 1.06–1.22) (Supplementary Figure S11).

## 4. Discussion

There is a rapidly growing interest in the prevention of cancer by modifying dietary structure and lifestyle. In this expanding meta-analysis of 41 observational studies, we focused on ovarian cancer risk related to both dietary fat intakes and serum lipids. The current results suggest four significant associations for dietary fat, including dietary total fat and cholesterol, saturated fat, and animal fat. Moreover, we found that higher serum triglyceride levels increased the risk of ovarian cancer. Furthermore, the significant associations between polyunsaturated and trans-fat and ovarian cancer risk were only observed in a case–control study. Our meta-analysis, which covered only prospective studies, provides new evidence on the relationships between dietary fats, serum lipid levels, and the risk of ovarian cancer and could strengthen its internal validity.

Our study suggested that dietary saturated fatty acids, trans-fatty acids, and animal-derived fats may be a risk factor for increased ovarian cancer development and that this positive association is biologically plausible. Foods high in saturated fats, especially processed and red meats, have been related to a higher risk of cancer and mortality (69). Nutritional factors, including trans-fatty acids, may be implicated in peroxisome proliferator-activated receptor (PPAR) γ stimulator. Elaic acid, a major long-chain trans-fatty acid, has been shown to transform cancer by altering specific G protein and epidermal growth factor receptor signaling pathways (70). Co-culturing adipocytes and ovarian cancer cells lead to the direct transfer of lipids from adipocytes to ovarian cancer cells, which promotes the growth of tumors both *in vitro* and *in vivo* (5). Obesity also increased lipogenesis, improved vascularity, and reduced M1 macrophage infiltration, all of which assisted ovarian cancer metastatic success (7).

Dietary cholesterol has always been controversial for its health effects. The marginal relationships between dietary cholesterol intake and the risk of ovarian cancer in our meta-analysis may have some biological basis. This finding was consistent with a meta-analysis of dose–response from seven studies, which found a marginally positive association (per 50 mg/day: RR = 1.01, 95% CI = 1.00–1.03) between dietary cholesterol intake and ovarian cancer risk (20). Cholesterol is a special lipid that is required for membrane biogenesis, cell proliferation, and differentiation. In addition to the dietary source, cholesterol can be synthesized in humans by the liver and carried throughout the body via the transporters LDL and HDL. Some studies have reported a positive association between serum cholesterol, a mandatory precursor of steroid hormones involved in cancer promotion and death (71). Experimental studies showed that high-level cholesterol could activate oncogenic effects by promoting systemic inflammation or directly binding to smoothened receptors, which in turn activate Hedgehog signaling and the mevalonate pathway (72–74). According to the available evidence, lipid-lowering medicines, such as statins, PARP inhibition, fenofibrate, and PCSK9, have been implied to have anti-tumor effects in several human malignancies, including ovarian cancer (68, 75, 76). The current meta-analysis demonstrated a positive association between serum triglycerides, but not cholesterol, and the risk of ovarian cancer development, with higher heterogeneity. Even so, we did not exclude the possibility that total serum cholesterol was etiologically significant in ovarian cancer. Therefore, it is necessary to investigate if serum lipid biomarkers can predict the risk of ovarian cancer.

However, this study also had several limitations that must be considered when interpreting the results. First, diet data in most of the published study were collected from a food frequency questionnaire or 24-h retrospective method by dietary recall or self-reported, which may cause failures in terms of memory and data inaccuracy and result in a significant bias. Second, individuals are mostly from North America and Europe, and the results of the meta-analysis may have implications for extrapolation to other regions due to differences in geographic location, ethnicity, and dietary patterns. Third, another limitation concerns the direct relationship between intakes of dietary fat and serum lipid levels because higher intakes of dietary fat do not necessarily lead to hyperlipidemia. Studies have now shown that some foods have the potential to improve lipid profiles, such as synbiotics (77) and probiotics (78) from yogurt or dairy. Several randomized controlled trials have also reported that improved dietary structure or food items can effectively improve serum lipid levels in patients with cardiovascular disease (77–79). However, no study has yet assessed both dietary fat intakes and serum lipids with ovarian cancer risk. In addition, due to the limited number of studies included for synthesis, we could not identify the association of the ratio of LDL-C to HDL-C with ovarian cancer risk, even though some studies reported that LDL/HDL can predict the occurrence of cardiovascular diseases (80).

In conclusion, the present up-to-date and expanding meta-analysis indicated that higher intake of dietary total fat, cholesterol, saturated fat, and animal fat was associated with an increased risk of ovarian cancer. Moreover, our evidence suggested that higher levels of serum triglycerides also increased the risk of ovarian cancer. Extensive prospective studies with more authentic measurements of nutritional or dietary variables are needed to confirm the significant association between serum dyslipidemias and ovarian cancer risk.

## 5. Conclusion

This meta-analysis indicated that higher daily intaker of total fat, saturated fat, animal fat, and cholesterol and higher levels of serum triglycerides were significantly associated with an increased risk of ovarian cancer.

## Author contributions

XZ, H-MD, and B-YL were all responsible for the conception and design of the manuscript. XZ and L-FD searched the literature, screened for final inclusion of the articles, completed the quality assessment, and planned the data analysis. XZ performed the meta-analysis. XZ, G-CC, and B-YL wrote the manuscript. XZ, G-CC, J-FL, LF, JL, Z-YH, FJ, Z-LZ, and B-YL revised and edited the manuscript. All authors contributed to the article and approved the submitted version.
