# Supplementary material for: Dietary fats and serum lipids in relation to the risk of ovarian cancer: a meta-analysis of observational studies
Source: Front Nutr. 2023 Sep 14;10:1153986. doi: 10.3389/fnut.2023.1153986 (PMC10538548; doi:10.3389/fnut.2023.1153986)
Supplement: Supplementary file 1 [file Data_Sheet_1.docx]

Supplementary Material

**Dietary fats and serum lipids in relation to risk of ovarian cancer：a meta-analysis of observational studies**

**Xu Zhang ^1^, †, Hong-Mei Ding ^2^, †, Li-Feng Deng ^3^, †, Guo-Chong Chen ^1^, Jie Li ^1^, Ze-Yin He ^1^, Li Fu ^2^, Jia- Fu Li ^4^, Fei Jiang ^4^, Zeng-Li Zhang ^4^, Bing-Yan Li ^1*^**

*** Correspondence:** bingyanli@suda.edu.cn Bing yan Li, Department of Nutrition and Food Hygiene, School of Public Health, Medical College of Soochow University, 199 Renai Road, Suzhou Industrial Park, Suzhou, 215123, China

**Supplementary Data**

**Supplementary Table S1.** Search strategy in PubMed, Web of Science, and Embase.

**Supplementary Table** **S1.** Search strategy in PubMed, Web of Science, and Embase

| **Database** | **Search strategy** |
| --- | --- |
| PubMed/MEDLINE. | *("Dietary Fats" OR "Fat, Dietary" OR "Cholesterol" OR "Triglycerides" OR*  *"Triacylglycerols" OR "Dyslipidemias" OR "Dyslipoproteinemias" OR "Lipoproteins, HDL" OR "High-Density Lipoprotein" OR "Lipoprotein, High Density" OR " Lipoproteins, LDL" OR "Low-Density Lipoproteins" OR "LDL-2" OR " LDL-1") AND ("Ovary " OR ovarian OR " ovary " OR*  *" Tumor " OR " Neoplasms " OR "Cancer")* |
| Scopus. Web of Science. | *("Dietary Fats" OR "Cholesterol" OR "Triglycerides" OR "Dyslipidemias" OR "Lipoproteins, HDL" OR " Lipoproteins, LDL" AND ("Ovary " OR " Tumor " OR " Neoplasms " OR "Cancer")*  *https://www.webofscience.com/wos/alldb/summary/2d2e0b72-411b-4a1d-824f-2249cd9200ad-46bdec52/relevance/1* |
| EMBASE Broad search | *'fat intake'/exp OR 'fat intake' OR 'cholesterol'/exp OR cholesterol OR 'triglycerides'/exp OR triglycerides OR 'dyslipidemias'/exp OR dyslipidemias OR 'high density lipoprotein cholesterol'/exp OR 'high density lipoprotein cholesterol' OR 'low density lipoprotein cholesterol'/exp OR 'low density lipoprotein cholesterol' AND 'ovary cancer'/exp OR 'ovary cancer'* |
